# Critical Intersectionality, Ageism, and Intergenerational Supports Foregrounding Black SGL and Gay Older Men

**DOI:** 10.1093/geroni/igaa057.3108

**Published:** 2020-12-16

**Authors:** Angela Perone, Beth Glover Reed, Danae Ross

**Affiliations:** 1 University of Michigan, Ypsilanti, Michigan, United States; 2 University of Michigan, Ann Arbor, Michigan, United States

## Abstract

Using critical intersectionality frameworks, this project foregrounds how Black same-gender-loving (SGL), gay, and bisexual older men navigate complexities of interacting positionalities (e.g. race, gender, sexual orientation, HIV-status, and class). This study employs and further develops intracategorical and intercategorical analytic methods with data from eight focus groups, conducted as part of a larger collaborative project in Detroit. Data from two intragroup focus groups with Black same-gender-loving older men and six subsequent intergroup focus groups with Black and white lesbian, gay, bisexual, SGL, and queer participants of various ages revealed concerns and responses to barriers and facilitators for intergenerational support and intergenerational transfer of knowledge. Building on intersectionality frameworks of power, this research provides new insights from a vastly underrepresented and understudied community about how shifting contexts shape how experiences of oppression like racism, ageism, and homophobia interact and reveal potential opportunities for intergenerational supports moving forward.

